# Comparison of Efficacy and Safety of Original and Biosimilar Adalimumab in Active Rheumatoid Arthritis in a Real-World National Cohort

**DOI:** 10.3390/medicina58121851

**Published:** 2022-12-15

**Authors:** Claudiu Costinel Popescu, Corina Delia Mogoșan, Luminița Enache, Cătălin Codreanu

**Affiliations:** 1 Rheumatology Department, “Carol Davila” University of Medicine and Farmacy, 020021 Bucharest, Romania; 2 “Dr. Ion Stoia” Clinical Center for Rheumatic Diseases, 020983 Bucharest, Romania

**Keywords:** rheumatoid arthritis, adalimumab, biosimilar, real-world data, national registry

## Abstract

*Background and Objectives:* Real-world evidence should reflect the evidence obtained from controlled trials; therefore, the study aimed to compare biosimilar adalimumab (bADA) to original adalimumab (oADA) in terms of efficacy and safety in a real-life national cohort of rheumatoid arthritis (RA) patients. *Materials and Methods*: The following study is a prospective observational study in which we analyzed patients treated with reimbursed biologics from the Romanian Registry of Rheumatic Diseases (RRBR). RA cases must fulfill the 2010 classification criteria, as well as specific inclusion and exclusion criteria. The RRBR database was queried for all RA patients starting oADA or bADA (FKB327, GP2017, MSB11022, SB5 available) from 2 May 2019 (the first bADA initiation) until 26 March 2022 (study search date). *Results:* The study included 441 patients who started oADA (48.3%) or bADA (51.7%) in the same time period. At baseline, patients starting bADA had a significantly higher mean age and lower prevalence of women. After the first six months of treatment, there were no significant differences between the oADA and bADA regarding rates of Boolean (15.0% vs. 12.3%, *p* = 0.401), DAS28-CRP (32.4% vs. 34.2%, *p* = 0.686) and SDAI (16.4% vs. 14.0%, *p* = 0.483) remission rates. There were 107 cases of adverse events (AE): 81.3% on oADA and 18.7% on bADA. Notably, 51.4% of AE were infections. Regarding severity, 49.5% of AEs were mild, 34.6% were moderate, and 15.9% were severe. *Conclusion:* Biosimilar adalimumab showed similar efficacy and safety to original adalimumab after the first six months of treatment in RA patients from a national registry.

## 1. Introduction

As soon as the diagnosis of rheumatoid arthritis (RA) is made, aggressive and tailored treatment of active synovitis aims to prevent and stop radiographic progression, control systemic inflammation, improve the patient’s quality of life, and prevent functional disability and early professional retirement. In the account of the above statements, the current target in RA treatment is the reaching of sustained Boolean or index-defined remission [1] or low disease activity (defined according to any of the validated composite disease activity scores) [2] if remission is not feasible, just like in the so-called “difficult-to-treat” patients [3,4]. In order to achieve this target [2], the treatment strategy currently and ideally employs combinations of conventional synthetic disease-modifying antirheumatic drugs (csDMARDs), especially methotrexate if not contraindicated or ineffective, with biologic DMARDs (bDMARDs) or with targeted synthetic DMARDs (tsDMARDs), and short term of low dose oral glucocorticoids (prednisone, methylprednisolone) for symptom control.

One of the most successful bDMARD for the treatment of active RA is original adalimumab, a fully human recombinant monoclonal antibody (immunoglobulin G1), derived by phage display, which targets with high affinity and specificity tumor necrosis factor α (TNFα) [5], a key cytokine in the pathogenic process of RA [6]. Adalimumab was first approved for RA in 2002 at a usual subcutaneous dose of 40 mg every two weeks. It has since proved its efficacy (versus placebo, using the American College of Rheumatology—ACR therapeutic response criteria) and safety [7,8,9,10,11] with high-quality evidence of meta-analyses of randomized trials. Its stable efficacy in obtaining therapeutic targets in RA made adalimumab a standard active comparator in clinical trials versus other bDMARDs and tsDMARDs [12,13,14,15].

Numerous adalimumab biosimilars have emerged [16] since the patent expiry of the original molecule in 2016, aiming theoretically to increase access to bDMARDs and to improve cost benefits (lower healthcare expenses, cost reduction by market competition). These new adalimumab molecules have since exhibited similar efficacy (judging by ACR-defined therapeutic responses) and safety in RA patients compared to the originator molecule in controlled clinical trials [17,18,19], even in clinical scenarios in which patients transitioned from original adalimumab to its biosimilar [20]. The way these findings mirror real-life clinical practice is highly heterogeneous, mainly since rheumatologists treat all referred patients (including “difficult to treat” cases), using less strict inclusion criteria, which vary by country depending on economic and accessibility factors, and since clinical decisions are largely based on DAS28 variance. Therefore, real-world evidence should complete the evidence obtained from controlled clinical trials [21] in different medical and economic scenarios.

In this context, the current study aims to compare the efficacy and safety profile of biosimilar adalimumab to those of original adalimumab in a real-life national cohort of RA patients.

## 2. Materials and Methods

### 2.1. Patients

This study derives from the analysis of the Romanian Registry of Rheumatic Diseases (RRBR) and is designed as a prospective observational study using a national electronic database that includes all RA patients treated with reimbursed modern molecules (bDMARDs and tsDMARDs). Efficacy and safety data are uploaded by each attending rheumatologist for each patient every 6 months, while safety data are uploaded at any time. Prior to treatment and inclusion in the RRBR, all patients gave written informed consent for both b/tsDMARD therapy and scientific use of their registry data. All RA patients fulfilled the 2010 classification criteria [22], as well as specific inclusion and exclusion criteria that have been published previously [23].

On 2 May 2019, the first Romanian RA patient received a form of biosimilar adalimumab; therefore, the database was queried for all cases of RA patients starting adalimumab from that date, with at least two complete visits (baseline and 6 months) until the study search date (26 March 2022). In this time frame, along with the original adalimumab molecule, there were 4 biosimilar adalimumab molecules available and actively prescribed in the country, as designated by their study names [24]: FKB327 [25,26,27], GP2017 [28,29,30], MSB11022 [31,32] and SB5 [33,34,35].

### 2.2. Variables

Data were retrieved electronically and retrospectively from the RRBR database and included demographics, RA phenotype characteristics, treatment, efficacy parameters, and safety data. Adalimumab initiation date was used to compute the age of the patients and the RA disease duration from the onset and from diagnosis. Sex was determined from the Romanian personal numerical code. Body mass index (BMI) was calculated as the ratio of weight in kilograms to square height in meters, and obesity was defined as BMI ≥ 30 kg/m^2^.

Serology status (rheumatoid factor—RF; anti-citrullinated protein antibodies—ACPA), inflammatory markers (C-reactive protein—CRP; erythrocyte sedimentation rate—ESR) and infection screening markers (anti-HBs antibodies, anti-hepatitis C virus antibodies, interferon-γ release assay) were recorded according to each local laboratory standards.

Tender and swollen joint counts (TJC, SJC) and RA bone erosions and ankyloses on conventional radiographs of hands and feet were reported by each attending physician. Patient (PtGA) and physician (PhGA) global assessments were measured on 100 mm visual analogue scales.

Disease activity score using 28 joints (DAS28) was calculated with 4 variables (TJC, SJC, PtGA, ESR, or CRP). Remission was defined as DAS28 < 2.6, low disease activity (LDA) as 2.6 ≤ DAS28 ≤ 3.2, moderate disease activity (MDA) as 3.2 < DAS28 ≤ 5.1, and high disease activity (HDA) as DAS28 > 5.1 [36,37]. Clinical Disease Activity Index (CDAI) [38] was calculated with 4 variables (TJC, SJC, PtGA, PhGA), and remission was defined as CDAI ≤ 2.8, LDA as 2.8 < CDAI ≤ 10, MDA as 10 < CDAI ≤ 22 and HDA as CDAI > 22. Simplified Disease Activity Index (SDAI) [39] was calculated with 5 variables (TJC, SJC, PtGA, PhGA, CRP), and remission was defined by SDAI ≤ 3.3, LDA as 3.3 < SDAI ≤ 11, MDA as 11 < SDAI ≤ 26 and HDA as SDAI > 26. Boolean remission was also assessed using the following simultaneous criteria: TJC ≤ 1, SJC ≤ 1, PtGA ≤ 1 cm, and CRP ≤ 1 mg/dL [1].

### 2.3. Statistics

Data distribution normality was assessed using descriptive statistics, normality, stem-and-leaf plots, and the Lillefors-corrected Kolmogorov–Smirnov tests. Continuous variables are reported as “mean ± standard deviation” if normally distributed or as “median (interquartile range)” if non-normally distributed, while dichotomous variables are reported as “observed frequency (percentage of subgroup).” In managing missing data, large fractions of missing cases from a specific variable led to it not being reported.

Independent-sample two-tailed *t*-tests (for normally-distributed data) and Mann–Whitney U tests (for non-normally distributed data) were used to assess differences in continuous variables among original and biosimilar adalimumab groups, while the associations of these subgroups with other categorical variables were studied using χ^2^ tests.

The statistical tests were considered significant if *p* < 0.05. All the statistical analysis and figures were performed using IBM SPSS Statistics version 25.0 for Windows (IBM Corp., Armonk, New York, NY, USA) and GraphPad Prism version 8.4.3 for Windows (GraphPad Software, San Diego, CA, USA).

## 3. Results

### 3.1. Sample

The RRBR database search for adalimumab treatment in RA revealed 1470 active cases, including 2 patients with unspecified bDMARDs and 12 patients on non-adalimumab bDMARDs, leaving 1456 RA patients on adalimumab (99.0%). The first biosimilar adalimumab was started on 2 May 2019, so all patients starting original adalimumab earlier were excluded (*n* = 1015), leaving 441 patients (30.3%) who started either original (*n* = 213; 48.3% of the analysis group) or biosimilar adalimumab (*n* = 228; 51.7%) in the same time period.

### 3.2. Baseline

The only notable baseline differences between patients on original adalimumab (*n* = 213) and patients on biosimilar adalimumab (*n* = 228) were the significantly higher mean age and disease duration to adalimumab initiation and, respectively, the lower prevalence of women in the latter group (Table 1).

The proportion of baseline missing data for some optional RRBR database variables was considered too high to accurately characterize the sample and therefore was not reported, namely the presence of at least one RA-specific bone erosion (157 missing cases, 35.6% of sample) and the presence of at least one RA-induced ankylosis (200 missing cases, 45.4%).

### 3.3. Efficacy after Six Months

Compared to patients starting original adalimumab, patients starting biosimilar adalimumab presented after the first six months of treatment significantly higher mean values of ESR and a non-significant lower prevalence of glucocorticoids (Table 2).

**Figure 1:**
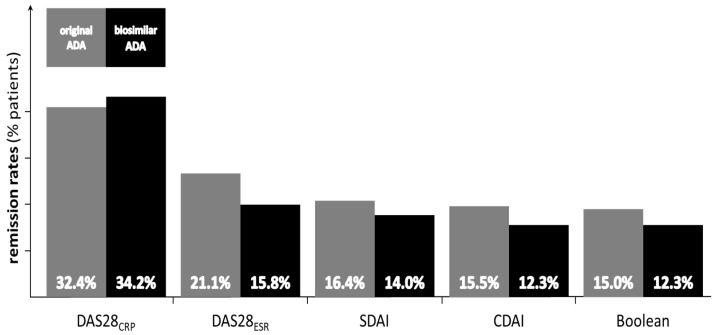
The 6-month remission rates of the 213 patients on original adalimumab and 228 on biosimilar adalimumab, according to the following definitions: DAS28CRP (*p* = 0.727), DAS28ESR (*p* = 0.148), SDAI (*p* = 0.483), CDAI (*p* = 0.329) and Boolean (*p* = 0.401).

The subgroup of patients on biosimilar adalimumab generally had a lower prevalence of composite score-defined remission, but the differences were not statistically significant regardless of the definition of remission (Figure 1).

**Figure 2:**
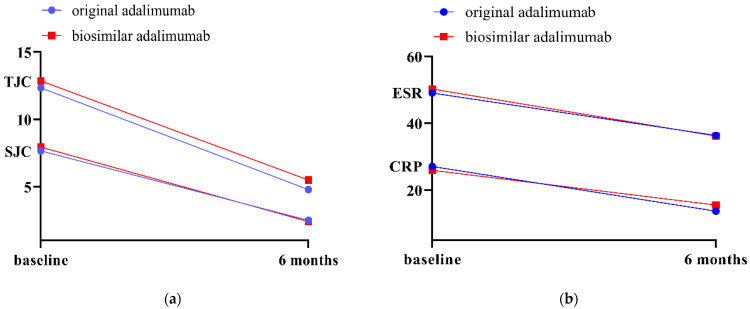
Grouped mean values of joint counts (**a**) and of inflammatory markers ((**b**); CRP in mg/L, ESR in mm/h, normalized by outlier exclusion for illustration purposes) at baseline and 6 months in patients starting either original (*n* = 213; blue circle) or biosimilar adalimumab (*n* = 228; red square).

Biosimilar adalimumab paralleled the amplitude of improvement, modeled by mean difference, of clinical examination parameters (TJC, SJC) and inflammatory markers (ESR, CRP) compared to original adalimumab (Figure 2).

### 3.4. Safety

The database search produced 300 adverse events (AE) cases, of which 57 appeared before the first biosimilar adalimumab, 124 cases were patients on current non-adalimumab b/tsDMARDs with no previous exposure to adalimumab, and in 12 cases, the current bDMARD was not recorded, and the previous bDMARD was either non-adalimumab or missing, leaving 107 cases of AE while on adalimumab (35.7%; Table 3).

Of these, 87 AE were reported while on current original adalimumab (81.3%), and 20 AE were reported while on current biosimilar adalimumab (18.7%). Notably, 51.4% of all recorded AE were infections. In terms of severity, 53 AEs were considered mild (49.5%), 37 were moderate (34.6%), and 17 were severe (15.9%). In terms of outcome, 13 AEs required hospitalization (12.1%), 1 induced disability (0.9%), 4 had been life-threatening (3.7%), and 2 resulted in death (1.9%).

## 4. Discussion

In summary, our real-life data show similar efficacy and safety of biosimilar adalimumab compared to original adalimumab in the RRBR cohort after the first six months of treatment. This similar efficacy was observed in the context of different RA populations at baseline in terms of demographic characteristics (the biosimilar subgroup was slightly older and contained more men, without identifiable confounders explaining this difference), but with comparable phenotype, disease activity indices, and csDMARD treatment schemes.

The pharmacologic effect of inhibiting TNFα-driven inflammation explains the similar composite score response after the first six months of treatment, while the fact that this occurs in real-life patients adds to the evidence supporting the role of biosimilar bDMARDs in the modern management of RA patients.

Surprisingly, the level of efficacy of both original and biosimilar adalimumab in this national RA cohort is concordant with the one reported by literature reviews [24,40] and by the randomized clinical trials which lead to the approval of different biosimilar adalimumab molecules in RA. For example, FKB327 was studied in 730 RA patients, resulting in the least squares mean DAS28-CRP at week 24 of 3.43 for FKB327 and 3.42 for original adalimumab [27]. In another study, 353 patients were randomized to either GP2017 or original adalimumab, reporting a time-weighted averaged change from baseline in DAS28-CRP until week 24 or −1.85 for GP2017 and −1.93 for original adalimumab (∆ = 0.08; 95% CI: −0.11–0.27) [29]. After 24 weeks of either MSB11022 or original adalimumab, the mean DAS28 was approximately 3.2 for both molecules in a study group of 288 patients [31]. Finally, the mean change from baseline to week 24 in the DAS28-ESR was comparable between SB5 and original adalimumab groups (−2.74 vs. −2.68) in a total sample of 542 RA patients [35].

This observation may be caused by the RA severity restriction, which applies both in randomized clinical trials and in national reimbursement criteria of bDMARDs: to begin bDMARD treatment, RA patients should have severe disease which failed to respond to csDMARDs, regardless of the definitions of severity and csDMARD-failure employed in both clinical settings.

In the national reimbursement scheme, the justification of severity restrictions is usually non-medical but cost-related, and biosimilars seem not to help in lowering the degree of required severity for bDMARD treatment: consulting the National Public Catalog of the maximum prices of medicines for human use (https://www.ms.ro/2021/03/19/in-atentia-dapp-reprezentanti-2/, accessed on 27 August 2022), containing the prices valid until 28 February 2023, at an exchange rate of 4.8734 Lei/Euro communicated by the National Bank of Romania on 26 August 2022, revealed that the rounded-up maximum price of a 40 mg prefilled pen of original adalimumab, including value-added tax, was 229 Euros, while the same price for four different biosimilar adalimumab products were respectively 204, 211, 226 and 229 Euros.

Another important observation is that, after six months of treatment, regardless of adalimumab type, a fraction of approximately 15% of patients are in Boolean, DAS28-ESR, SDAI, and CDAI remission, and a fraction of approximately 33% of patients are in DAS28-CRP remission. While the fact that DAS28-CRP performs better could be explained by the large interval of possible values of CRP and the relative speed with which it decreases after anti-inflammatory stimuli (bDMARDs, glucocorticoids), the fact remains that remission is a rare state in the treatment of RA, even in the age of bDMARDs.

Considering the short follow-up period and the relatively small sample size, safety data regarding both AE and severe AE do not differ significantly among patients treated with original and biosimilar adalimumab in the RRBR database and are comparable to literature reports [24]. If they are related to the pharmacological effect of the molecule, these AE are generally both explained by either the effects of TNFα inhibition in physiological immune responses (i.e., infection risk manifested in this RRBR cohort, for example by one case each of lower respiratory tract infection) or by an immune response to the therapeutic protein itself (i.e., hypersensitivity manifested in this RRBR cohort, for example by one case each of anaphylaxis).

There are several study limitations that can influence the relevance of the observed results, which we discussed above. RRBR’s design requires data input from multiple users (approximately 300 rheumatologists in the country), which can generate variability in data entry. Although RRBR can capture data regarding comorbidities, imaging efficacy endpoints, quality of life, general health status (HAQ, EQ-5D), and radiographic progression, their fields are not mandatory for data input, and this results in a very high proportion of missing data. In the RRBR cohort, immunogenicity assessments are not done. Further directions may address these issues and expand the analysis to greater observation intervals and to other biosimilar bDMARDs and other indications, such as spondyloarthritis and psoriatic arthritis.

## 5. Conclusions

Biosimilar adalimumab showed similar efficacy and safety to original adalimumab after the first six months of treatment in RA patients with high disease activity from a national registry (RRBR), which brings further evidence for biosimilarity in patients in a real-world setting.

## Figures and Tables

**Table 1:** Baseline demographic and RA data.

	ADA (*n* = 441)	oADA (*n* = 213)	bADA (*n* = 228)	*p*
women	79.8%	86.9%	73.2%	0.000
age (birthdate-ADA; y)	57 ± 13	55 ± 13	58 ± 12	0.015
DD (onset-ADA; y)	10.1 ± 8.3	9.3 ± 7.4	10.9 ± 9.0	0.064
DD (diagnosis-ADA; y)	8.7 ± 7.7	8.1 ± 7.1	9.2 ± 8.2	0.122
urban dwelling	64.2%	66.7%	61.8%	0.291
BMI (kg/m^2^)	27.0 ± 5.3	26.9 ± 5.5	27.2 ± 5.2	0.540
obesity	25.4%	25.4%	25.4%	0.983
smoking	12.2%	14.6%	10.1%	0.153
employed	28.1%	30.5%	25.9%	0.279
university education	20.6%	19.2%	21.9%	0.487
RF +	86.8%	88.2%	86.3%	0.559
ACPA +	74.1%	83.0%	78.3%	0.223
RF and ACPA +	71.2%	72.8%	69.7%	0.482
HBs antigen +	2.3%	2.3%	2.2%	0.913
anti-HCV antibodies +	1.6%	1.9%	1.3%	0.637
IGRA test +	15.2%	13.6%	16.7%	0.372
b/tsDMARDs naïve	57.8%	59.6%	56.1%	0.459
oral glucocorticoids	34.7%	34.7%	34.6%	0.984
no csDMARDs	3.3%	4.2%	1.8%	0.125
1 csDMARD	62.4%	60.6%	64.0%	0.452
>1 csDMARD	33.3%	35.2%	34.2%	0.825
methotrexate	57.8%	48.8%	42.1%	0.157
ESR (mm/h)	50 ± 26	48 ± 25	51 ± 27	0.144
CRP (mg/L)	17.5 (28.2)	17.9 (29.2)	16.9 (26.2)	0.450
DAS28CRP	5.5 ± 1.3	5.5 ± 1.3	5.5 ± 1.2	0.792
DAS28-CRP remission	3.6%	3.8%	3.5%	0.890
DAS28-CRP LDA	1.6%	2.3%	0.9%	0.217
DAS28-CRP MDA	23.4%	24.4%	22.4%	0.612
DAS28-CRP HDA	71.4%	69.5%	73.2%	0.382

+ positive; ACPA—anti-citrullinated protein antibodies; b/oADA biosimilar/original adalimumab; b/tsDMARDs—biologic/targeted synthetic disease-modifying antirheumatic drugs; BMI—body mass index; CRP—C-reactive protein; DD disease duration; ESR—erythrocyte sedimentation rate; HB—hepatitis B; HCV—hepatitis C virus; HDA—high disease activity; LDA—low disease activity; MDA—moderate disease activity; RF—rheumatoid factor; y years.

**Table 2:** Efficacy after the first six months of adalimumab.

	ADA (*n* = 441)	oADA (*n* = 213)	bADA (*n* = 228)	*p*
glucocorticoids	7.9%	10.3%	5.7%	0.072
no csDMARDs	4.3%	6.1%	2.6%	0.073
1 csDMARD	71.2%	70.0%	72.4%	0.576
>1 csDMARD	24.5%	23.9%	25.0%	0.797
methotrexate	44.7%	47.9%	41.7%	0.189
ESR (mm/h)	36 ± 28	33 ± 25	39 ± 30	0.028
CRP (mg/L)	5.5 (13.2)	6.0 (14.8)	4.7 (10.8)	0.208
Boolean remission	13.6%	15.0%	12.3%	0.401
DAS28-CRP	3.5 ± 1.5	3.3 ± 1.6	3.5 ± 1.5	0.727
DAS28-CRP remission	33.3%	32.4%	34.2%	0.686
DAS28-CRP LDA	20.2%	21.1%	19.3%	0.633
DAS28-CRP MDA	28.3%	27.2%	29.4%	0.616
DAS28-CRP HDA	18.1%	19.2%	17.1%	0.559
DAS28-ESR	4.1 ± 1.6	4.1 ± 1.6	4.2 ± 1.6	0.607
DAS28-ESR remission	18.4%	21.1%	15.8%	0.148
DAS28-ESR LDA	13.4%	11.7%	14.9%	0.328
DAS28-ESR MDA	42.2%	39.0%	45.2%	0.187
DAS28-ESR HDA	26.1%	28.2%	24.1%	0.333
SDAI	15.9 ± 14.4	16.1 ± 14.2	15.7 ± 14.6	0.756
SDAI remission	15.2%	16.4%	14.0%	0.483
SDAI LDA	34.7%	33.3%	36.0%	0.562
SDAI MDA	27.7%	25.4%	29.8%	0.294
SDAI HDA	22.4%	24.9%	20.2%	0.236
CDAI	14.5 ± 13.2	14.7 ± 13.2	14.3 ± 13.2	0.734
CDAI remission	13.8%	15.5%	12.3%	0.329
CDAI LDA	36.7%	34.7%	38.6%	0.401
CDAI MDA	27.0%	25.4%	28.5%	0.455
CDAI HDA	22.4%	24.4%	20.6%	0.339

b/oADA biosimilar/original adalimumab; CDAI—clinical disease activity index; CRP—C-reactive protein; csDMARDs—conventional synthetic disease-modifying antirheumatic drugs; DAS—disease activity score; DD disease duration; ESR—erythrocyte sedimentation rate; HDA—high disease activity; LDA—low disease activity; MDA—moderate disease activity; SDAI—simplified disease activity index; y years.

**Table 3:** Number of reported AEs in RA patients while on adalimumab.

	ADA	oADA	bADA		ADA	oADA	bADA
	(*n* = 107)	(*n* = 87)	(*n* = 20)		(*n* = 107)	(*n* = 87)	(*n* = 20)
*infection*				*skin*			
pulmonary TB	1	1	0	rash	4	4	0
HBV infection	1	1	0	CSD	1	1	0
UTI	16	15	1	psoriasis	1	1	0
pneumonia	6	4	2	vasculitis	1	1	0
URTI	10	9	1	eczema	2	1	1
COVID19	12	8	4	*lung*			
otitis media	1	1	0	ILD	1	1	0
enterocolitis	2	1	1	RA nodules	1	1	0
skin ulcer	2	1	1	*other AE*			
cutaneous HZ	1	0	1	pregnancy	1	1	0
septic arthritis	1	1	0	anxiety disorder	1	1	0
conjunctivitis	2	1	1	anemia	1	1	0
*cancer*				CKD	2	2	0
solid tumor	1	1	0	fracture	3	2	1
blood cancer	2	2	0	R’sP	1	1	0
*cardiovascular*				MAS	1	0	0
ACS	1	1	0	IVDH surgery	3	1	2
stroke	2	2	0	hip arthroplasty	1	1	0
acute DVT	1	1	0	cataract surgery	2	0	2
CAD	1	1	0	hypothyroidism	3	3	0
AHT	3	2	1	transaminitis	1	1	0
*metabolic*				anaphylaxis	2	1	1
dyslipidemia	4	4	0	death	2	2	0
hyperuricemia	1	1	0	unspecified AE	1	1	0

ACS—acute coronary syndrome; AE—adverse event; AHT—arterial hypertension; b/oADA—biosimilar/original ADA; CAD—coronary artery disease; CKD—chronic kidney disease; CSD—chronic spongiotic dermatitis; DVT—deep vein thrombosis; ENT—ear-nose-throat; HBV—hepatitis B virus; HZ—herpes zoster; ILD—interstitial lung disease; IVDH—intervertebral disk hernia; MAS—macrophage activation syndrome; oADA—original ADA; R’sP—Raynaud’s phenomena; TB—tuberculosis; URTI—upper respiratory tract infection; UTI—urinary tract infection.

## Data Availability

The data presented in this study are available on request from the corresponding author. The data are not publicly available due to patient confidentiality.
